# Hindfoot Valgus following Interlocking Nail Treatment for Tibial Diaphysis Fractures: Can the Fibula Be Neglected?

**DOI:** 10.1155/2014/806363

**Published:** 2014-12-07

**Authors:** Metin Uzun, Adnan Kara, Müjdat Adaş, Bülent Karslioğlu, Murat Bülbül, Burak Beksaç

**Affiliations:** ^1^Orthopaedic Department, Acıbadem Maslak Hospital, Darüşşafaka Street, Büyükdere Street No. 40, Maslak, Sarıyer, Istanbul, Turkey; ^2^Orthopaedic Department, Şişli Etfal Training and Education Hospital, Şişli, Istanbul, Turkey; ^3^Okmeydani Education and Training Hospital, Okmeydani, Istanbul, Turkey; ^4^Orthopaedic Department, Kasımpaşa Military Hospital, Istanbul, Turkey; ^5^Orthopaedic Department, Medipol University, Istanbul, Turkey; ^6^Orthopaedic Department, Acıbadem University, Darüşşafaka Street, Büyükdere Street No. 40, Maslak, Sarıyer, Istanbul, Turkey

## Abstract

*Purpose*. We evaluated whether intramedullary nail fixation for tibial diaphysis fractures with concomitant fibula fractures (except at the distal one-third level) managed conservatively with an associated fibula fracture resulted in ankle deformity and assessed the impact of the ankle deformity on lower extremity function. *Methods*. Sixty middle one-third tibial shaft fractures with associated fibular fractures, except the distal one-third level, were included in this study. All tibial shaft fractures were anatomically reduced and fixed with interlocking intramedullary nails. Fibular fractures were managed conservatively. Hindfoot alignment was assessed clinically. Tibia and fibular lengths were compared to contralateral measurements using radiographs. Functional results were evaluated using the Knee Injury and Osteoarthritis Outcome Score (KOOS) and the Foot and Ankle Disability Index Score (FADI). *Results*. Anatomic union, defined as equal length in operative and contralateral tibias, was achieved in 60 fractures (100%). Fibular shortening was identified in 42 fractures (68%). Mean fibular shortening was 1.2 cm (range, 0.5–2 cm). Clinical exams showed increased hindfoot valgus in 42 fractures (68%). The mean KOOS was 88.4, and the mean FADI score was 90. *Conclusion*. Fibular fractures in the middle or proximal one-third may need to be stabilized at the time of tibial intramedullary nail fixation to prevent development of hindfoot valgus due to fibular shortening.

## 1. Introduction

Diaphyseal tibial fractures are generally treated surgically and are frequently accompanied by fibular fractures [1–3]. The usual treatment for a concomitant fibular fracture in the distal one-third is surgical fixation, but if it occurs in the middle or proximal one-third, it is typically treated symptomatically [1–3]. Ankle alignment and functional results in patients symptomatically treated for fibular fractures are unclear [1, 2, 4, 5].

We evaluated whether conservative treatment for these concomitant-associated fibular fractures leads to ankle deformity and assessed the functional results of an ankle deformity, if present.

## 2. Materials and Methods

**Figure 1:**
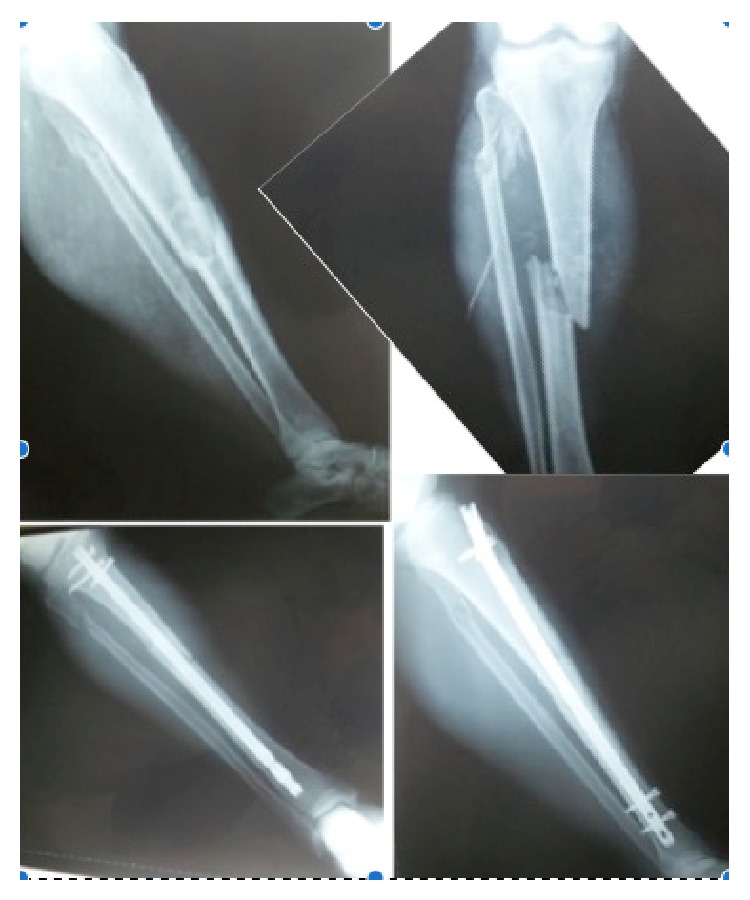
Anteroposterior and lateral X-ray images showing a middle one-third tibial shaft fracture with an associated fibular fracture and a healed tibial fracture treated with intramedullary nailing.

The study included 60 consecutive patients with unilateral middle one-third tibial fractures treated by anatomic reduction and interlocking intramedullary nail fixation and who had been managed conservatively for a proximal to middle one-third (except distal one-third level) fibular fracture. Three surgeons performed the surgeries at three different centers. All fractures were closed. Patients who had tibial union complications and/or who had undergone a second surgery were excluded. The mean follow-up period was 20 months (range, 12–36 months) (Figure 1).

**Figure 2:**
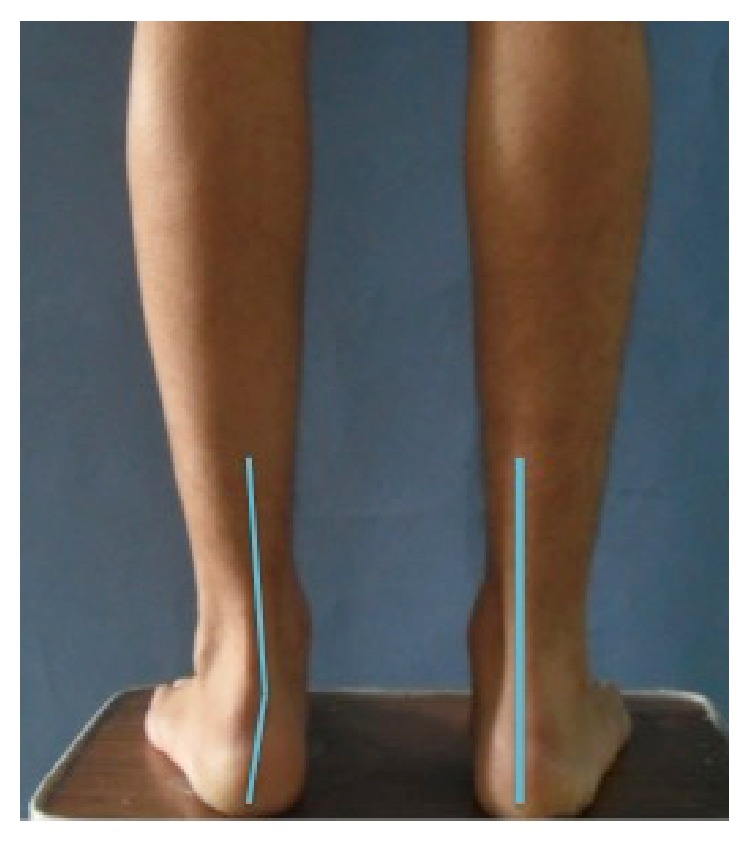
Stance position. Solid lines were marked on the skin, and dashed lines have been added to the photograph to identify the tibial angle and the calcaneal stance.

Hindfoot alignment was evaluated clinically with a goniometer at the follow-up assessment, in the manner described by Astrom and Arvidson [6] (Figure 2). Full-length anteroposterior and lateral radiographs of the tibia and fibula were obtained for both lower extremities at the same distance and used to compare tibial and fibular lengths for the injured and contralateral limbs [6]. Lengthening was measured with a computer digital system. Functional outcome scores were determined for each patient using the Knee Injury and Osteoarthritis Outcome Score (KOOS) and the Foot and Ankle Disability Index Score (FADI) [7, 8].

This study was approved by the authors' Institutional Review Board. All authors certify that their institution has approved the reporting of this case series and that all investigations conformed with the ethical principles of research.

## 3. Results

**Figure 3:**
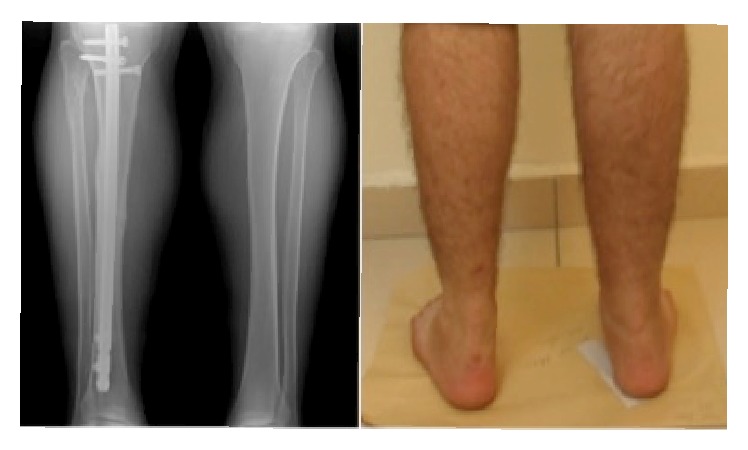
Clinical images showing the crus heel relationship.

**Figure 4:**
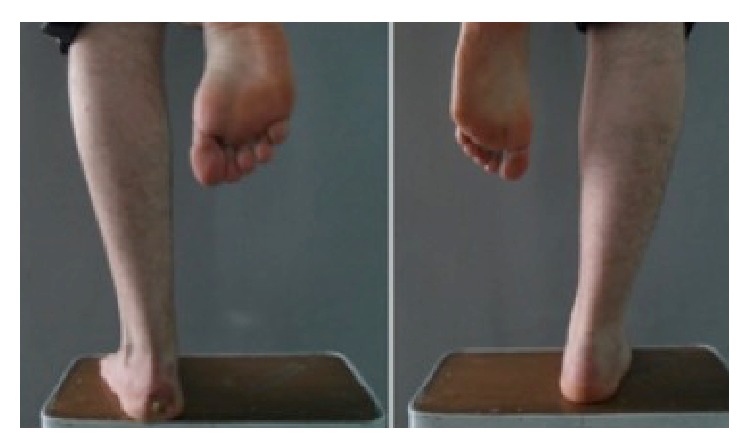
Clinical images showing differences in opposite extremities due to weight-bearing.

All patients demonstrated radiographic union of both the tibia and fibula. Anatomic union occurred in 60 cases (100%) and was defined for tibia with an equal length to the contralateral limb (Figure 1). Fibular shortening was identified in 42 cases (68%). Mean fibular shortening was 1.2 cm (range, 0.5–2 cm) (Figure 3). The increase in calcaneal valgus on the clinical hindfoot examination was a mean of 5° compared to that of the other extremity (Figures 3 and 4). The mean KOOS score was 88.4, and the mean FADI score was 90.

## 4. Discussion

Tibial fractures are one of the most frequent orthopedic problems [1, 3]. Treatment remains controversial due to the variety of fracture patterns and soft tissue problems associated with these injuries.

Successful results have been reported for tibial intramedullary nailing in cases with associated proximal and middle one-third fibular fractures, as in the current study [9–11]. Although associated distal one-third fibular fractures are less commonly present with tibial diaphysis fractures, the accepted treatment for these cases is surgical fixation of the fibula. Associated distal one-third fibular fractures were excluded from our study, but we evaluated outcomes from nonsurgical management for more proximal fibular fractures associated with tibial shaft fractures treated with intramedullary fixation. Complications have been reported following both surgical and conservative treatment of tibial fractures, including lower extremity angulation, rotational deformity, and ankle malalignment [12, 13]. Although these complications may compromise lower extremity function and result in knee or ankle pain, impaired tibial alignment was noted, but no complications related to the ankle were reported in a study by Pobłocki et al. [14]. In contrast to those results, a previous study on patients with tibial fractures found that a delayed diagnosis was associated with a Maisonneuve, syndesmotic, or posterior or medial malleolar fracture at a rate of 20.1% [10]. A tibial alignment disorder may be a rather common complication for distal or proximal fractures, and functional impairment is expected due to the long-term effects of an alignment disorder, but a hindfoot deformity usually does not occur following a middiaphyseal fracture.

Although fibular fractures may lead to fibular shortening, Hooper et al. [2] reported successful results after conservatively treating isolated diaphyseal fibular fractures. No complications related to the ankle occurred but no information was provided about bone shortening. In the present study, fibular shortening was common, and a valgus calcaneal deformity was noted after bone healing from a tibial shaft fracture. Fibular length was measured and compared to a healthy fibula using the same exposure time, position, and distance on X-rays. Although computed tomography is the best method for measuring fibular length, it was not available to us. A hindfoot assessment must be done in a standing, weight-bearing position. We used the goniometer method described by Astrom and Arvidson [6]. Coronal plane alignment can be assessed using X-rays as described by Saltzman [15] or on a long axial view under weight-bearing. We did not use these techniques, as X-ray quality depends on the technician [15, 16]. A hindfoot alignment disorder is dynamic; thus, hindfoot cases must be evaluated in the weight-bearing position both clinically and radiologically.

Whorton and Henley [11] showed that fixing the fibula in patients with open tibial and fibular shaft fractures has no effect on bone healing or alignment of the tibia. However, some studies have reported that fixing the fibula in patients with distal tibial or fibular fractures increases fracture stability up to week 12 after surgery [16–18]. Biomechanical results have shown that fixing the fibula may help prevent loss of alignment in distal tibial fractures repaired with either intramedullary nailing or plate fixation methods [16, 19].

## 5. Conclusion

Shortening the fibula in patients with upper level fibular fractures associated with tibial fractures treated with intramedullary nailing may cause a dynamic hindfoot valgus deformity. All hindfoot cases must be evaluated in the weight-bearing position both clinically and radiologically. Additional study is needed to determine if long-term functional outcomes in patients treated with tibial nailing can be improved by surgical reduction and fixation of associated suprasyndesmotic fibular fractures.

## References

[1] Garrick J. G., Riggins R. S., Requa R. K., Lipscomb P. R. (1972). Fracture of the mid-shaft of the tibia and fibula. A survey of treatment. *Clinical Orthopaedics and Related Research*.

[2] Hooper G., Buxton R. A., Gillespie W. J. (1981). Isolated fractures of the shaft of the tibia. *Injury*.

[3] Keating J. F., Kuo R. S., Court-Brown C. M. (1994). Bifocal fractures of the tibia and fibula. Incidence, classification and treatment. *Journal of Bone and Joint Surgery*.

[4] Weise K., Hermichen H., Weller S. (1985). Significance of the fibula in tibial fractures and pseudarthroses. *Aktuelle Traumatologie*.

[5] Merchant T. C., Dietz F. R. (1989). Long-term follow-up after fractures of the tibial and fibular shafts. *The Journal of Bone and Joint Surgery—Series A*.

[6] Astrom M., Arvidson T. (1995). Alignment and joint motion in the normal foot. *Journal of Orthopaedic & Sports Physical Therapy*.

[7] Roos E. M., Roos H. P., Lohmander L. S., Ekdahl C., Beynnon B. D. (1998). Knee injury and osteoarthritis outcome score (KOOS)—development of a self-administered outcome measure. *Journal of Orthopaedic & Sports Physical Therapy*.

[8] Martin R. L., Burdett R. G., Irrgang J. J. (1999). Development of the foot and ankle disability index (FADI). *Journal of Orthopaedic & Sports Physical Therapy*.

[9] Schmidt A. H., Finkemeier C. G., Tornetta P. (2003). Treatment of closed tibial fractures. *Instructional Course Lectures*.

[10] Stuermer E. K., Stuermer K. M. (2008). Tibial shaft fracture and ankle joint injury. *Journal of Orthopaedic Trauma*.

[11] Whorton A. M., Henley M. B. (1998). The role of fixation of the fibula in open fractures of the tibial shaft with fractures of the ipsilateral fibula: Indications and outcomes. *Orthopedics*.

[12] Vallier H. A., Cureton B. A., Patterson B. M. (2012). Factors influencing functional outcomes after distal tibia shaft fractures. *Journal of Orthopaedic Trauma*.

[13] Cannada L. K., Anglen J. O., Archdeacon M. T., Herscovici D., Ostrum R. F. (2009). Avoiding complications in the care of fractures of the tibia. *Instructional Course Lectures*.

[14] Pobłocki K., Domaradzki M., Gawdzik J., Prochacki P., Rajewski R. (2011). Complications after intramedullary nailing of the tibia. *Chirurgia Narządów Ruchu i Ortopedia Polska*.

[15] Saltzman C. L., El-Khoury G. Y. (1995). The hindfoot alignment view. *Foot & Ankle International*.

[16] Strauss E. J., Alfonso D., Kummer F. J., Egol K. A., Tejwani N. C. (2007). The effect of concurrent fibular fracture on the fixation of distal tibia fractures: a laboratory comparison of intramedullary nails with locked plates. *Journal of Orthopaedic Trauma*.

[17] Kumar A., Charlebois S. J., Cain E. L., Smith R. A., Daniels A. U., Crates J. M. (2003). Effect of fibular plate fixation on rotational stability of simulated distal tibiae fractures treated with intramedullary nailing. *Journal of Bone and Joint Surgery—Series A*.

[18] Egol K. A., Weisz R., Hiebert R., Tejwani N. C., Koval K. J., Sanders R. W. (2006). Does fibular plating improve alignment after intramedullary nailing of distal metaphyseal tibia fractures?. *Journal of Orthopaedic Trauma*.

[19] Stufkens S. A., van Bergen C. J., Blankevoort L., van Dijk C. N., Hintermann B., Knupp M. (2011). The role of the fibula in varus and valgus deformity of the tibia: a biomechanical study. *Journal of Bone and Joint Surgery*.

